# Antioxidant Capacity of Preterm Neonates Assessed by Hydrogen Donor Value

**DOI:** 10.3390/medicina55110720

**Published:** 2019-10-30

**Authors:** Melinda Matyas, Monica G. Hasmasanu, Gabriela Zaharie

**Affiliations:** 1 Neonatology Department, University of Medicine and Pharmacy, Cluj-Napoca, 400006, str. V. Babes no. 8, Romania; monica.hasmasanu@gmail.com (M.G.H.); gabrielazaharie1966@gmail.com (G.Z.); 2 1st Neonatology Department, County Emergency Hospital Cluj-Napoca, 400006, str. Clinicilor no. 3–5, Romania

**Keywords:** oxidative stress, premature, respiratory distress, antioxidant defense

## Abstract

*Background and objectives:* Premature newborns have a number of oxidative stress-inducing disorders. Antioxidant defense is deficient in premature newborns. Hydrogen donors can be used to evaluate the non-enzymatic antioxidant defense. By measuring hydrogen donors, a group of antioxidants can be assessed: tocopherol, ascorbic acid, and glutathione. These represent the most relevant group of non-enzymatic antioxidants. The main aim of this study was to evaluate the non-enzymatic antioxidant defense capacity of premature newborns by measuring hydrogen donors. *Materials and Methods:* We evaluated the non-enzymatic antioxidant capacity by hydrogen donor measurement in 24 premature newborns with various oxidative stress-inducing disorders and in 14 premature newborns without oxidative stress-inducing conditions. Statistical analysis was performed using the Statistica program (v. 8, StatSoft, Round Rock, TX, USA). Differences between groups were tested with Wilcoxon matched test for quantitative paired data or Mann–Whitney test for quantitative independent data. The Z test for proportions was used to compare qualitative data among subgroups. *Results:* Hydrogen donors in the study group had a significantly lower value on the first day of life compared to the value of the control group. Also, the hydrogen donor value in the study group was significantly lower on the first day compared to the third day of life (*p* < 0.05). Neonates with mild respiratory distress (14 cases) had increased hydrogen donor values on their third day of life compared to the first day of life. *Conclusions:* The antioxidant capacity is influenced by oxidative stress-inducing disorders. Respiratory distress influenced the hydrogen donor value and antioxidant defense. Antioxidant defense gradually improves after birth according to gestational age.

## 1. Introduction

Antioxidant defense mechanisms are divided into two main categories: enzymatic and non-enzymatic. Hydrogen donors evaluate the non-enzymatic antioxidant defense capacity. Cysteine, glutathione, ascorbic acid, tocopherol, polyphenols, aromatic amines, and protein sulfhydryl (SH) groups are common natural non-enzymatic antioxidants. Hydrogen donor capacity evaluation is based on the reduction of the stable free radical by a number of its non-enzymatic antioxidant components: glutathione, tocopherol, and ascorbic acid. SH groups in the umbilical cord blood at neonates are reduced because of their low protein level. The plasma protein level is closely correlated with gestational age without the presence of the same correlation between gestational age and sulfhydryl groups; this is due to a change in the level of sulfated amino acids according to diet and under influence of different diseases. Sulfhydryl groups are significantly affected in transfused patients. Oxidative stress represents an imbalance between free radical production and the capacity of antioxidant defense mechanisms to neutralize free radicals [1,2,3,4].

Antioxidant defense mechanisms develop in the last trimester of pregnancy. Therefore, premature newborns have a high risk of oxidative stress. This risk is generated by the premature birth itself, by the diseases associated with preterm birth (e.g., respiratory distress, intraventricular hemorrhage, or necrotizing enterocolitis), as well as a deficient antioxidant defense mechanism (induced by prematurity) [1,5,6,7]. The “free radical disease” described by Saugstad [8] includes several disorders such as bronchopulmonary dysplasia (BPD), patent ductus arteriosus, necrotizing enterocolitis, retinopathy of prematurity (ROP), hypoxic–ischemic encephalopathy, and cerebral hemorrhage. Hyperbilirubinemia contributes to the increase of the antioxidant defense capacity and the maturation of antioxidant enzymes [3,9]. The slow increase of bilirubin values in patients with circulatory insufficiency, sepsis, and asphyxia might be explained by exhaustion in the context of increased oxidative stress in these disorders [4].

The aim of this study was to evaluate antioxidant activity using the hydrogen donor capacity of premature newborns with asphyxia, respiratory distress syndrome (RDS), and/or cerebral hemorrhage (as oxidative stress-generating disorders), compared to premature newborns without any oxidative stress-generating disorders.

## 2. Materials and Methods

A non-randomized prospective observational study was conducted in the Neonatology Department of the County Emergency Clinical Hospital Cluj-Napoca from January 2007 to October 2009. The study was approved by the Ethical Committee of the university, no.68/23.05.2006.

The study group was represented by premature newborns with different complications related to preterm birth that generate oxidative stress such as respiratory distress, asphyxia, cerebral hemorrhage. The diagnosis of respiratory distress due to surfactant deficiency was established based on clinical signs: tachypnea, grunting, increased work of breathing, cyanosis, pallor, lethargy, apnea, and radiological criteria (e.g., reticulogranular pattern and air bronchograms). Respiratory distress was quantified in three severity forms based on clinical criteria, the Silverman score. Asphyxia at birth was defined by Apgar score below 3 at 5 min and pH < 7 at birth. The diagnosis of cerebral hemorrhage was established by head ultrasound. The control group consisted of late preterm newborns, with a gestational age of 35–36 weeks, without any of the pathologies of the study group. 

Premature newborns with hemolytic disease, malformations, chromosomal diseases, as well as those who died in the first two days of life were not included in the study. 

### Blood Sample 

The quantification of the hydrogen donor capacity was done on the first and third days of life for preterm neonates, while for controls, only one determination was carried out on the first day of life. The measurement of the serum hydrogen donor capacity was based on the reduction of the stable radical 1,1-diphenyl-picrylhydrazyl (DPPH) by a number of its non-enzymatic antioxidant components: glutathione, cysteine, tocopherol, and ascorbic acid. This reduction can be traced as a color change from violet to pale yellow, evidenced by an alteration of absorbance at 520 nm. 

For hydrogen donor measurement, the serum samples obtained after centrifugation of whole blood samples were diluted with a 10 mM phosphate buffer, pH 7.4. Then, the 0.1 mM DPPH solution was added, and the samples were left for 30 min at room temperature. The extinction was read compared to blank samples which consisted of samples treated in the same way, but to which absolute methanol was added instead of the DPPH solution. In parallel, the extinction of control samples that did not contain serum but a corresponding volume of phosphate buffer was determined.

The hydrogen donor capacity was measured as inhibition % compared to control samples, according to the following calculation formula [7]:

Inhibition % = (Extinction control − Extinction serum sample)/Extinction control × 100 (1)

Measurements were performed on a UV-VIS ABL JASCO 6000 double-fascicle spectrophotometer (Jasco, Tokyo, Japan).

Blood gas parameters such as pH, pCO_2,_ pO_2_, and basal excess were monitored from capillary blood samples using the machine of the unit Microgas 7650 (Radiometer, Basel, Switzerland).

Statistical analysis was performed using the Statistica program (v. 8, StatSoft, Round Rock, TX, USA). The results are presented as median and interquartile range defined as Q1–Q3 for quantitative data, and number and ratio for qualitative data. Differences between groups were tested with Wilcoxon matched test for quantitative paired data or Mann–Whitney test for quantitative independent data. The Z test for proportions was used to compare qualitative data among subgroups. Association analysis was done with Fisher’s exact test on r by c contingency tables or by Spearman’s rank correlation coefficient for paired data which did not respect the normal distribution. All comparisons were done at a significance level of 5% and all *p*-values smaller than 0.05 were considered statistically significant.

The lab tests were performed after the parents’ informed consent was obtained.

## 3. Results

### 3.1. Characteristic of the Groups

The study group included 24 premature newborns with a gestational age between 28 and 36 weeks, and 14 controls without significant differences between genders. Half of the subjects (12/24) in the case group had a gestational age less than or equal to 32 weeks. The control group comprised 14 newborns with a median of gestational age equal to 36 weeks (IQR = (35–36) and equal number of girls and boys) (7/7). The body weight of cases proved significantly lower as compare to controls (case vs. control: 1550 g (1375 to 1900) vs. 2300 g (2250 to 2400), Mann–Whitney test: Z-stat = −4.41, *p* < 0.0001). Furthermore, the Apgar score proved significantly lower among cases as compared to controls (case vs. control: 10 (10 to 10) vs. 8 (7 to 9), Mann–Whitney test: Z-stat = −4.49, *p* < 0.0001).

No significant differences between genders were observed among preterm infants with complications in terms of gestational age and birth weight, but the Apgar score had a tendency to significantly differ, with higher values among boys compared to girls (Table 1).

One patient had hypoxic–ischemic encephalopathy (1/24).

### 3.2. Blood Gas Parameters

Table 2 presents the dynamics of the blood gas parameters in preterm newborns with complications. With the exception of pCO_2_, the values of blood gas parameters proved significantly higher at the second determination compared to the first determination. 

### 3.3. Hydrogen Donors 

The values of hydrogen donors measured on the first and third day significantly correlate to each other (Spearman’s rank correlation coefficient: *ρ* = 0.8185, *p* < 0.0001). A similar pattern was also observed for FiO_2_ (Spearman’s rank correlation coefficient: *ρ* = 0.5104, *p* = 0.0128).

The value of hydrogen donors (HDs) in the control group was 53.90 (48.60–56.90). The day 1 HD value for the study group was significantly lower compared to controls (Mann–Whitney *U* test: statistic = −2.58, *p* = 0.0100).

Hydrogen donors had significantly lower values on the first day compared to the third day among patients with mild respiratory distress (Table 3). In cases with severe respiratory distress, the trend of the HD value was the same as in the mild form of the disease, but the difference was not statistically significant (*p* > 0.05). This can be due to the small number of cases with moderate RDS. In the moderate form of RDS, we found a minor decrease in HD value, but we have to consider that only 3 patients had this form of disease.

The values of HD determined on the first day were significantly lower among those with cerebral hemorrhage compared to those without cerebral hemorrhage (Table 4). The values of HD on the third day significantly increased among those with cerebral hemorrhage (Table 4). This increasing trend in HD values on the third day compared to the first day of life was maintained in preterm neonates without cerebral hemorrhage, but the difference was not statistically significant. However, it should be considered that only 5 premature newborns in the study group had cerebral hemorrhage.

No significant association was observed between the FiO_2_ and HD values for the study group (measurements on the first day: Spearman’s rank correlation coefficient: *ρ* = 0.3510, *p* = 0.0926; measurements on the third day: Spearman’s rank correlation coefficient: *ρ* = 0.0930, *p* = 0.6655). The median HD value for controls was 53.90 [48.60 to 56.90]. On the first day, the median HD in controls was significantly higher than in the study group (*p* < 0.05) (Table 5).

HD value according to the Apgar score at 5 min is presented in Table 6. No association was found between the Apgar score and HD value.

## 4. Discussions

In premature newborns, susceptibility to oxidative stress is higher due to their immaturity, with increased energy requirements that exceed their aerobic cellular metabolism capacity to the reduced homeostatic mechanism activation capacity and deficiencies in antioxidant defense that mature gradually in the first year of life [7,10,11]. Reactive oxygen species scavengers, such as ceruloplasmin, transferrin, or lactoferrin, are also underdeveloped in preterm neonates [12,13,14].

Our study evaluated the non-enzymatic antioxidant capacity of premature newborns with various oxidative stress-generating diseases associated with preterm birth. Hydrogen donors assess a large group of natural non-enzymatic antioxidants: cysteine, glutathione, ascorbic acid, tocopherol, polyphenols, aromatic amines, and protein sulfhydryl (SH) groups [14,15].

The hydrogen donor value of the study group was higher on the third day compared to the first day (*p* < 0.05) (Table 2). The pH value showed the same evolution (*p* = 0.0046). The improvement of the antioxidant capacity with the postnatal age of newborns in the case group can be explained by the improvement of the oxidative stress-generating pathology associated with prematurity (asphyxia, RDS). The antioxidant capacity improves with postnatal age through a decrease of free radical production with the improvement of oxidative stress-generating disorders, as well as due to the improvement of antioxidant defense systems. Similar findings are described by Perrone et al. [9] in their study, which reports the loss of glutathione caused by a reduced capacity of cellular conservation at a low gestational age. Hyperoxia is associated with low cysteine levels in neonates with respiratory distress. Cysteine has a key role in glutathione synthesis [16].

The significant correlation of HD values (*p* < 0.05) with the RDS was demonstrated for the mild form of disease. There were no similar findings for the medium form of RDS. In severe forms, there was an improvement in HD value on the third day of life. The majority of the study group (75%) had an improved HD value on the third day of life. RDS contributes to free radical production, which puts stress on the deficient antioxidant capacity of premature newborns. There was no significant correlation between HD and FiO_2_ values for the study group. Oxygen exposure was controlled, avoiding hyperoxia. Hyperoxia represents an important factor that increases oxidative stress. The FiO_2_ value decreased on the third day of life in the study group (*p* = 0002). The role of RDS in decreasing the total antioxidant capacity is described by Negi et al [17] in their study on 16 preterm newborns with RDS.

In the study group, cerebral hemorrhage is another factor that enhances free radical production and oxidative stress. Although there are few cases, the HD value is significantly lower in newborns with cerebral hemorrhage compared to those without cerebral hemorrhage (*p* < 0.05). The low value of HD in cases with cerebral hemorrhage it could be related to the oxidative stress and consumption of antioxidants in this condition. Cerebral hemorrhage is one of the disorders included by Saugstad in the definition of “free radical disease” and is a factor that increases oxidative stress in newborns [8,18].

In the neonates of the control group, the HD value was significantly higher compared to that of premature newborns with various associated diseases, as has been reported in other studies [6,9]. The antioxidant defense capacity is higher as the gestational age increases. In the control group, there were no oxidative stress-generating disorders (RDS, asphyxia, cerebral hemorrhage) that influence the deficient antioxidant capacity [13,19]. Oxidative stress-inducing disorders determine a decrease in antioxidant capacity [20]. As a result, hydrogen donors have lower values in preterm newborns with oxidative stress-generating diseases such as RDS, cerebral hemorrhage, or asphyxia at birth.

While other studies demonstrated a correlation between HD values and asphyxia, our study failed to show any correlation. This could be explained by the small number of patients with asphyxia in this study [9,10].

Despite the relatively small size of the sample, our findings are in agreement with those reported by similar studies. However, further studies in larger groups are required to draw well-grounded conclusions and to allow using HD value as a means to evaluate the risk of diseases in premature newborns. The study group birth weight was >1375 g and gestational age (GA) of 32.5 weeks. This age category is not at highest risk of developing ROP, BPD, a severe form of RDS, and intraventricular hemorrhage. This represent a limitation of the study. However, all patients of the study group had an oxidative stress-generating condition. In the control group, there was no exposure to oxidative stress-generating factors. Also, the GA in control was ≥34 weeks. The differences of GA and non-exposure to oxidative stress can explain the difference between the HD values of the two groups. Along with total antioxidant capacity, hydrogen donors are used as a method for the evaluation of antioxidant defense mechanisms. The method used in this study is relatively easy to use. It allows for both the evaluation of the antioxidant defense of preterm newborns and assessment of its evolution based on the observed antioxidant deficit. In addition, it can be used in the selection of additional therapeutic methods that limit oxidative distress and improve/maintain the antioxidant defense of preterm newborns. Very few studies related to oxidative stress in newborns in our geographical region have been published.

## 5. Conclusions 

Hydrogen donor values in premature newborns are influenced by RDS or cerebral hemorrhage compared to preterm newborns without “free radical disease” disorders. The majority of neonates with RDS in the study group have improved HD values on their third day of life. 

Cerebral hemorrhage influenced the HD values in our study group. Overall, the antioxidant defense of preterm newborns is significantly reduced if any oxidative stress-generating condition is associated with preterm labor. Our data suggest that determination of the hydrogen donor capacity can be used as a marker of non-enzymatic antioxidant capacity in premature newborns with different pathologies. Considering the relatively small size of the study group, more extensive studies are required to support the results. Despite the relatively small size of the group, this study supports the findings of other studies according to which antioxidant defense in premature newborns is affected by oxidative stress-generating diseases associated with small gestational age.

## Figures and Tables

**Table 1:** Characteristics of the case group by gender.

Characteristic	All (n = 24)	Girls (n = 14)	Boys (n = 10)	Stat (*p*-Value)
Gender ^a^	24	14	10	−1.59 (0.1123)
Gestational age, weeks ^b^	32.5 (31 to 35)	33 (31.25 to 34.75)	32 (31 to 34.25)	0.61 (0.5387)
Weight, g ^b^	1550 (1375 to 1900)	1500 (1405 to 1890)	1650 (1365 to 1875)	0.00 (0.9999)
Apgar 1 min ^b^	7 (6 to 8)	6 (3 to 7)	7 (7 to 9)	−1.76 (0.0790)
Apgar 5 min ^b^	8 (7 to 9)	7 (6.25 to 8)	8.5 (8 to 9)	−1.73 (0.0841)
Respiratory distress ^c^ mild moderate severe	15/24 3/24 4/24	9/14 1/14 2/14	6/10 2/10 2/10	n.a. (0.8209)
Intraventricular hemorrhage ^c^	5/24	2/14	3/10	n.a. (0.6146)

^a^ no, Z-test for proportion; ^b^ median (Q1–Q3), Q = quartile, Mann–Whitney test; ^c^ no/total number of cases, Fisher’s exact test.

**Table 2:** Dynamics of blood gas parameters among preterm infants with complications.

Blood Gas Parameters	1st Day	3rd Day	Stat (*p*-Value)
FiO_2_ (n = 24)	40.00 ^b^ (35.00 to 42.50)	25.00 ^b^ (24.50 to 30.00)	3.74 (0.0002)
pH (n = 24)	7.30 ^b^ (7.30 to 7.34)	7.35 ^b^ (7.32 to 7.35)	2.84 (0.0046)
pCO_2_ (n = 24)	38.00 ^b^ (35.00 to 40.50)	35.00 ^b^ (30.90 to 40.00)	1.48 (0.1396)
pO_2_ (n = 24)	49.75 ^b^ (45.00 to 50.55)	55.00 ^b^ (55.00 to 58.15)	2.94 (0.0033)
SaO_2_ (n = 24)	80.00 ^b^ (80.00 to 90.00)	90.00 ^b^ (85.75 to 93.50)	3.68 (0.0002)
HD (n = 24)	43.75 ^b^ (39.95 to 52.28)	49.10 ^b^ (44.83 to 56.09)	2.39 (0.0170)

^b^ median (Q1–Q3), Q1 = first quartile, Q3 = third quartile; Wilcoxon matched test; FiO_2_ = oxygen %; pH = blood gas status; pCO_2_ = CO_2_ pressure; pO_2_ = oxygen pressure; SaO_2_ = oxygen saturation; HD = hydrogen donor

**Table 3:** Hydrogen donors by severity of respiratory distress and comparisons with the controls.

	1st Day	3rd Day	Stat (*p*-Value)
Mild (n = 15) Stat (*p*-value) *	42.20 ^b^ (39.80–45.30) −3.11 (0.0019)	46.65 ^b^ (41.53–52.05) −2.09 (0.0369)	2.42 (0.0157)
Moderate (n = 3) Stat (*p*-value) *	62.70 ^b^ (59.40–64.20) 1.55 (0.1218)	61.50 ^b^ (60.43–64.35) 1.68 (0.0926)	0.00 (0.9999)
Severe (n = 4) Stat (*p*-value) *	48.30 ^b^ (46.48–51.31) −1.02 (0.3082)	49.60 ^b^ (45.30–54.00) −0.91 (0.3650)	0.00 (0.9999)

^b^ median (Q1–Q3), Q = quartile, Wilcoxon matched test; * as compared to control.

**Table 4:** Hydrogen donors and cerebral hemorrhage among patients of case group.

Cerebral Hemorrhage	1st Day	3rd Day	Stat (*p*-Value) *
Absent (n = 19)	56.10 ^b^ (47.30–62.70)	59.36 ^b^ (47.20–61.50)	0.40 (0.6858)
Present (n = 5)	42.40 ^b^ (39.80–47.30)	49.00 ^b^ (43.75–52.50)	2.74 (0.0062)
Stat (*p*-value) **	−2.31 (0.0209)	−1.07 (0.2863)	

^b^ median (Q1–Q3), Q = quartile; * Mann–Whitney test; ** Wilcoxon matched test.

**Table 5:** Hydrogen donors (HD) evolution at case vs control.

	Group (n)	Median (Q1 to Q3)	Z-Value-Mann-Whitney test (*p*-Value)
Control (n = 14)	HD	53.90 (48.60 to 56.90)	
Case (n = 24)	HD 1	43.75 (39.95 to 52.28)	−2.56 (0.0104)

**Table 6:** HD according to Apgar score.

		HD DOL1	Apgar 5	Weight (g)
Case	Median	43.75	8	1550
	Q1	39.95	7	1375
	Q3	52.28	9	1900
		45.82	7	1591
		10.36	2	393
